# Actinomycete strain type determines the monodispersity and antibacterial properties of biogenically synthesized silver nanoparticles

**DOI:** 10.1186/s43141-021-00153-y

**Published:** 2021-04-16

**Authors:** Mostafa Mabrouk, Tarek A. Elkhooly, Shaimaa K. Amer

**Affiliations:** 1 Refractories, Ceramics and Building Materials Department, National Research Centre, 33El Bohouth St. (former EL Tahrir St.),- Dokki, Giza, P.O.12622 Egypt; 2 Biochemistry Department, Faculty of Medicine, Delta University for Science and Technology, Mansoura, Gamasa, Egypt; 3 Microbiology Department, Faculty of Science, Ain Shams University, Cairo, Egypt

**Keywords:** Actinomycete, Bio-nanotechnology, Low cost, AgNPs

## Abstract

**Background:**

Bio-nanotechnology is considered as one of the low-cost approaches that have been utilized in production of nanomaterials. The current research aimed at investigating the influence of different types of Actinomycete strains on the final properties of silver nanoparticles (AgNPs) such as size, shape, polydispersity, and antibacterial properties. For this purpose, the following techniques were employed UV spectrophotometer, SDS-PAGE electrophoresis, TEM, FTIR, antibacterial agar diffusion test, and Zetasizer.

**Results:**

It was found that among 34 Streptomyces isolates collected from the soil, *Streptomyces spiralis* and *Streptomyces rochei* were able to reduce silver nitrate into sliver nanoparticles. The diversity and molecular weights of extracellular proteins secreted by these stains were different as proved by SDS-PAGE technique. This consequently resulted in differences in polydispersity of AgNPs which indicate that the sizes of AgNPs were highly dependent on the amount, molecular sizes, and diversity of extracellular matrix proteins of the microorganism.

**Conclusion:**

This article might give an insight about the importance of molecular sizes of biomacromolecules such as proteins on the physical properties of biogenic synthesized nanoparticles.

## Background

The expression of bio-nanotechnology has been originated for nanotechnology field recently [1]. In bio-nanotechnology, nanomaterials are either prepared in the intracellular matrix of biological organisms or by using their extracts then, they can be applied in biological applications. It is thought that this technique has superiority features over the other classical and advanced techniques due to low cost, large-scale production and simplicity along with bio safety [2, 3]. Nevertheless, the biosynthesized possess have some restrictions owing to the difficulty of potent strain recognizing, storing of individual species in a sterile conditions and probabilities of infection and contaminations, especially nanomaterials prepared by bacterial species [4]. This motivated huge number of scientists to replace using bacterial species with other alternatives such as extract of plants and microorganisms like fungi and actinobacteria [5]. A green approach to fabricate nanoparticles has been evolved as a revolutionary discipline. Silver nanoparticles were extracellularly synthesized using *Aspergillus flavus* [6]. The synthesis of silver nanoparticles and its antioxidant, antimicrobial, and cytotoxic activities was investigated and extracellularly biosynthesized using *Penicillium italicum* isolated from Iraqi lemon fruits [7]. Promising green approach for the preparation of heterogeneous catalytic nanoparticles by investigating the synergistic effect of metal ions in spinel structure to enhance their photocatalytic activity was early reported [8]. Zinc oxide nanoparticles have been synthesized via the green and easy sol-gel method [9].

CoMnCrO4 nanoparticles were prepared via green sol–gel method by using the natural gel [10]. A green method to synthesize Fe oxide NPs using *Punica granatum* peel extract was also reported. The formation of Fe oxide NPs was optimized using different concentrations of peel extract (20 mL, 40 mL, and 60 mL) to achieve small size and better morphology [11]. The reduction of gold ions into gold nanoparticles using *Nerium oleander* leaf extract in the one step green synthetic method. The gold nanoparticles synthesized by this method were pure and showed good antioxidant activity [12]. The gold decorated titanium dioxide (Au/TiO_2_) nanocomposite was synthesized by a novel and ecofriendly green deposition method using phytochemicals from the aqueous extract of *Ranunculus muricatus* [13]. A novel and eco-friendly green procedure was developed for the synthesis of Au nanoparticles using stem extract of *Salvadora persica* [14]. Very few studies have been reported on actinobacteria; especially *streptomyces spiralis* being capable of synthesizing nanoparticles [15]. Actinobacteria are gram-positive filamentous bacteria which are widely found in both terrestrial and marine environment and have long been exploited commercially as an amusing source of unique secondary metabolites, such as antibiotics, enzymes, and enzyme inhibitors [16, 17]. In addition, they were recognized as an efficient possibility for metal nanoparticles synthesis creation extracellularly and intracellularly. Formulation of nanoparticles by actinomycetes possesses impressive polydispersity and stability. Furthermore, they have demonstrated significant biocidal activity against various microorganisms [18, 19].

Practically, it was confirmed that bioactive molecules such as exopolysaccharides are delivered by a variety of living beings either prokaryotes or eukaryotes including microorganisms, plants, and animals. Among these living beings, actinomycetes produce huge amounts of exopolysaccharides [20]. In bio-nanotechnology it is thought that the exopolysaccharides produced by actinomycetes have great influence on the distribution particle size and morphology. Actinomycetes are known to transform metal salts to metal nanoparticles extracellularly. Nevertheless, their synthesis mechanism of nanoparticles differs relying upon the strain type. The intracellularly produced metallic nanoparticles could in the end be effluxed out of the cell [21]. The extracellular blend of metal nanoparticles is commonly done by the reductive ingredients delivered from the cell. For instance, DNA, NADH-subordinate reductase, and sulphur-contained protein secreted from the cell can instigate the transform of Ag^+^ particle to Ag^0^ stage prompting the arrangement of silver nanoparticles (AgNPs) [22–24]. Since the critical features of metal nanoparticles are size-and shape-subordinate, the control of size and state of the particles is one of the most significant perspectives for their applications, especially biomedical usage [25]. Additionally, past examinations have announced that antimicrobial nature of AgNPs demonstrated to be relying upon their size and shape, where littler nanoparticles showed better antimicrobial activity [24]. It has been shown that AgNPs with various sizes and shapes can be produced utilizing different strain type [26]. Therefore, the present work is focused on the biosynthesis of silver nanoparticles utilizing actinobacteria and to elucidate its antimicrobial activity against pathogenic microorganisms. Moreover, the effect of utilized strain type is to be stressed out on the morphology, size, and polydispersity of the produced AgNPs, by UV spectrophotometer, PCR, antibacterial test, TEM, Zetasizer, and FTIR investigation techniques.

## Methods

### Collection of soil samples and isolation of Streptomycetes strains

Soil samples were collected from Halayeb and Shalatene along Red Sea Coast. The samples were collected in sterile polythene bags from the depth of 10–15 cm. Then, the samples were dried at room temperature and stored in sterile container for further process [27]. Thirty-four streptomycete isolates were collected from soil samples by serial dilution method [28] using starch nitrate medium. Predetermined amount (0.1 mL) of inoculum of the proper dilution was placed on each plate. The plates were incubated at 28 °C for 7 days. Streptomycetes were isolated according to their specific morphological features and then it was purified.

### Biosynthesis of silver nanoparticles

A loop full of each isolate was cultured into the flask (100 mL) containing 50 mL sterile starch nitrate broth and incubated in the shaker incubator at 150 rpm at 28 °C. The cells were harvested by centrifugation (at 6000 rpm for 10 min) and the supernatant (5 mL) was challenged with 5 mL of 1 mM silver nitrate solution (Sigma-Aldrich, USA) [29]. The tubes were incubated in shaker incubator (at 200 rpm) at 28 °C under dark conditions. During 48 h, the biosynthesis of silver nanoparticles was evaluated by transforming the suspension color into yellow to brown [30]. Two controls were done for every assessment of silver nanoparticles biosynthesis one of them containing 10 mL starch nitrate broth and the other contain 5 mL silver nitrate broth and 5 mL of 1 mM silver nitrate solution. The silver biosynthesis was detected every 24 h during 7 days. Silver reduction by Streptomyces was determined by change of colour from pale yellow to brown. In order to assess the effect of extracellular matrix proteins on size, shape, and polydispersity of silver nanoparticles, the bacterial broth containing AgNPs was reduced in the volume and the proteins in the extracellular matrix were concentrated by using an Amicon Ultra centrifugal filter with a 10 kDa cut-off (Merck Millipore; Darmstadt, Germany).

### UV spectrophotometer

Solutions of AgNPs were examined by UV–visible spectroscopy, were carried out in a UV–vis spectrophotometer (Jasco, V-730, Japan), operating in a wavelength from 200 to 900 nm.

### Growth curve of selected Streptomyces isolates

A growth curve was built to assess growth time of both strains of Streptomyces isolates and relate it with the production period of the silver nanoparticles. The isolates were grown in submerged culture for 10 days. The growth medium in one flask was filtered through a Whatman no. 1 filter paper, every 24 h within 10 days of growth period. Dry weight of cell mass was set up on the contrast between the final weight of the filter paper, after filtration and drying and the initial weight [31].

### Molecular identification of Streptomyces isolates and phylogenetic analysis

The genomic DNA was extracted from the cultures of two Streptomyces isolates by using kit (MicroSeq 500 16S rDNA bacterial identification Kits) according to the manufacturer’s instructions. Amplification of 16S rDNA by PCR was done using universal bacterial primer forward F (5′-CGGGCGGTGTGTAC-3′) and reverse R (5′-CAGCCGCGGTAATAC-3′) which amplify a ~ 800 bp. Amplification was carried out in a final volume of 50 μL containing; PCR buffer (1×), Taq DNA polymerase (2.5 U), dNTPs (4 mM), primers (0.4 μM), and template DNA (4 ng) with 100 bp ladder DNA marker. The thermal cycle (PCR) steps were applied as follows: 5 min initial denaturation at 95 ^o^C, followed by 30 cycles of 1 min denaturation at 95 ^o^C, 1 min primer annealing at 55 ^o^C, 1 min extension at 72 ^o^C and a final 10 min extension at 72 ^o^C. The amplified DNA fragment was separated on 1% (w/v) agarose gel electrophoresis, using TBE buffer containing ethidium bromide (1 μg/mL). A single ~ 800 bp DNA fragment was cut and extracted from the gel, using a Core Bio Gel Extraction Kit. The sequence was determined by the CinnaGen Company. Sequence data of partial 16S rDNA was aligned and analyzed for finding the closest homologous bacteria. The 16S rRNA nucleotide sequence was compared to nucleotide databases using BLASTN program that is available from the National Center for Biotechnology Information (NCBI, 2014) and retrieved ligned using GeneDoc software version 2.6. 002. Phylogenetic trees were built utilizing the neighbor-joining.

Screening for the extracellular proteins attached to AgNPs using SDS-PAGE.

To examine the diversity of extracellular proteins involved in Ag NPs synthesis and secreted from both Streptomyces strains in their numbers and molecular sizes, electrophoretic analysis of concentrated proteins on 12% SDS-PAGE was used. Briefly, after 48 h of adding the extract of the two Streptomyces strains that has been grown for 8 days to silver nitrate, the media of both strains were compiled and concentrated utilizing Amicon Ultra centrifugal filter with cut-off of 10 KDa. The proteins that are larger than 10 KDa was retained and concentrated in the upper part of the filter and their concentration was assessed by using Bradford assay. The extracellular proteins that are associated with Ag NPs was diluted in phosphate buffer saline, pH 7.2 and then 20 μg of the proteins of each strain was added to the wells of 12% polyacrylamide gel containing 10% Sodium dodecyl sulphate (SDS). A low molecular weight marker (Pharmacia Fine Chemicals Company) was used to show the distribution of molecular sizes of extracellular proteins associated with silver nanoparticles. Proteins on the gel were stained with 0.25% Coomassie Brilliant Blue R-250

### Particle size and distribution by TEM

Transmission electron microscope (TEM) was utilized to examine the effect of the strain type on the morphology, diameter, and distributions of synthesized AgNps. Practically, copper grid was submersed in each solution of AgNPs samples and allowed to dry in room temperature. The dried AgNPs were then characterized by TEM instrument (JEOL, JEM2100, Electron Microscope, TEM-HR, Japan).

### Absorption spectra of the AgNPs

Infrared absorption spectroscopy (FTIR) estimations were completed independently for each treatment to discover the compound that interferes in the preparation of AgNPs. Spectra of AgNPs were recorded after mixing with potassium bromide (1:100), which were introduced in pellets form. They were obtained by Broker Tensor 27 spectrophotometer. The spectra measuring range was 400–4000 cm^−1^.

### Antibacterial activity of AgNPs against pathogenic microorganisms

Antimicrobial activity of the AgNPs containing filtrate was done using the well diffusion method [32]. Antibacterial property of biosynthesized AgNPs was assessed against gram-positive and gram-negative pathogenic bacteria by well diffusion method. Bacterial strains used in this study were (*Pseudomonas aeruginosa* ATCC9027, *Escherichia coli* ATCC8739, *Salmonella enterica* ATCC14028, *Staphylococcus aureus* ATCC6538, *Bacillus subtilis* ATCC6633). Each microorganism was inoculated onto Muller-Hinton broth (Merck, Germany) and incubated at 37 °C in a shaker incubator at 200 rpm for 24 h. The fresh cultures were streaked onto the Muller-Hinton agar and wells (6 mm in diameter) were made in the medium using sterile sharp borer. Then 100 μL of each AgNPs were added into the wells. All plates were incubated at 37 °C for 24 h. Afterward, the clear zone of the growth inhibition for each AgNPs was measured and recorded [33].

### Particle size and charges of AgNPs in aqueous medium

Colloidal solutions of AgNPs were investigated by Zetasizer nano-series (Nano ZS), Malvern, UK, to assess their particle distribution, sizes and charges. Practically, AgNPs was suspended in distilled water and underwent to Zetasizer measurement. Simultaneously, the particle charges of AgNPs were also determined.

### Statistical analysis

In this work, all the obtained experimental data are expressed as the mean ± standard deviation (SD) for *n* = 3. The antibacterial results were analyzed analysed by Student’s *t* test, significance was set at *p* ˂ .0.05

## Results

### Molecular identification of Streptomyces isolates by 16S rRNA gene sequence analysis and phylogenetic analysis of Streptomycetes isolates

**Fig. 1:**
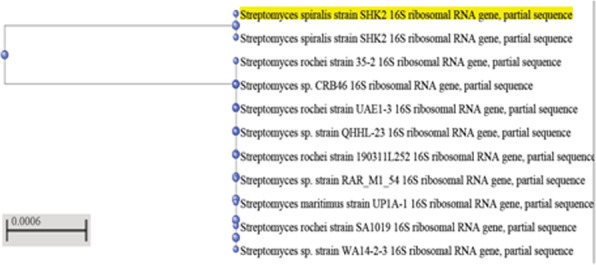
Phylogenetic analysis of Streptomycete isolate 5 using neighbour joining

**Fig. 2:**
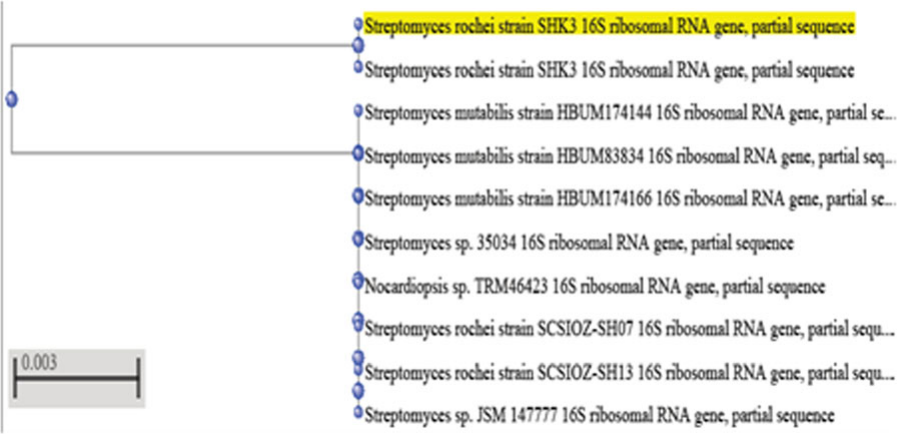
Phylogenetic analysis of Streptomycete isolate 7 using neighbour joining

The amplified DNA products separated on agarose gel electrophoresis revealed a single band of about 800 bp are illustrated in Fig. 1. PCR sequences of two streptomycete isolates were compared with the other sequenced streptomycetes in the NCBI and the database showed similarity with some sequences of other streptomycete upon molecular characterization of 16S rDNA gene sequence. The sequence data indicated that isolate 5 showed 99% similarity with *Streptomyces spiralis*, while isolate 7 exhibited 99–100% similarity with the genus *Streptomyces rochei*. The DNA sequences were published in the NCBI databases under the following specific accession numbers: 5 (MK559584) and 7 (MK559585). Phylogenetic analysis of the 16S rRNA sequence data showed that the streptomycete isolates 5 and 7 belonged to the genus *Streptomyces spiralis* and *Streptomyces rochei*, respectively, as shown in Figs. 1 and 2.

### Actinobacteria isolation and biosynthesis of AgNPs

The current study was focused on the extracellular synthesis of AgNPs from actinobacteria supernatant. A total of 34 actinomycetes isolates were isolated from collected soil sample from Halayeb and Shalatene area checked for their ability to produce AgNPs. The screening revealed that only two isolates numbers 5 and 7 identified as *Streptomyces spiralis* and *Streptomyces rochei*, respectively, showed the ability to synthesis AgNPs. The Ag^+^ ions reduction was evidently noticeable when AgNO_3_ was added to the supernatant of actinobacteria, and the color changed from yellow to dark brown. In the control, there was no color development. The maximum production of silver nanoparticles was observed at day 8 as will be explained in the following sections. Further increase in incubation time resulted in gradual decrease in production of nanoparticles.

### UV spectrophotometer

**Fig. 3:**
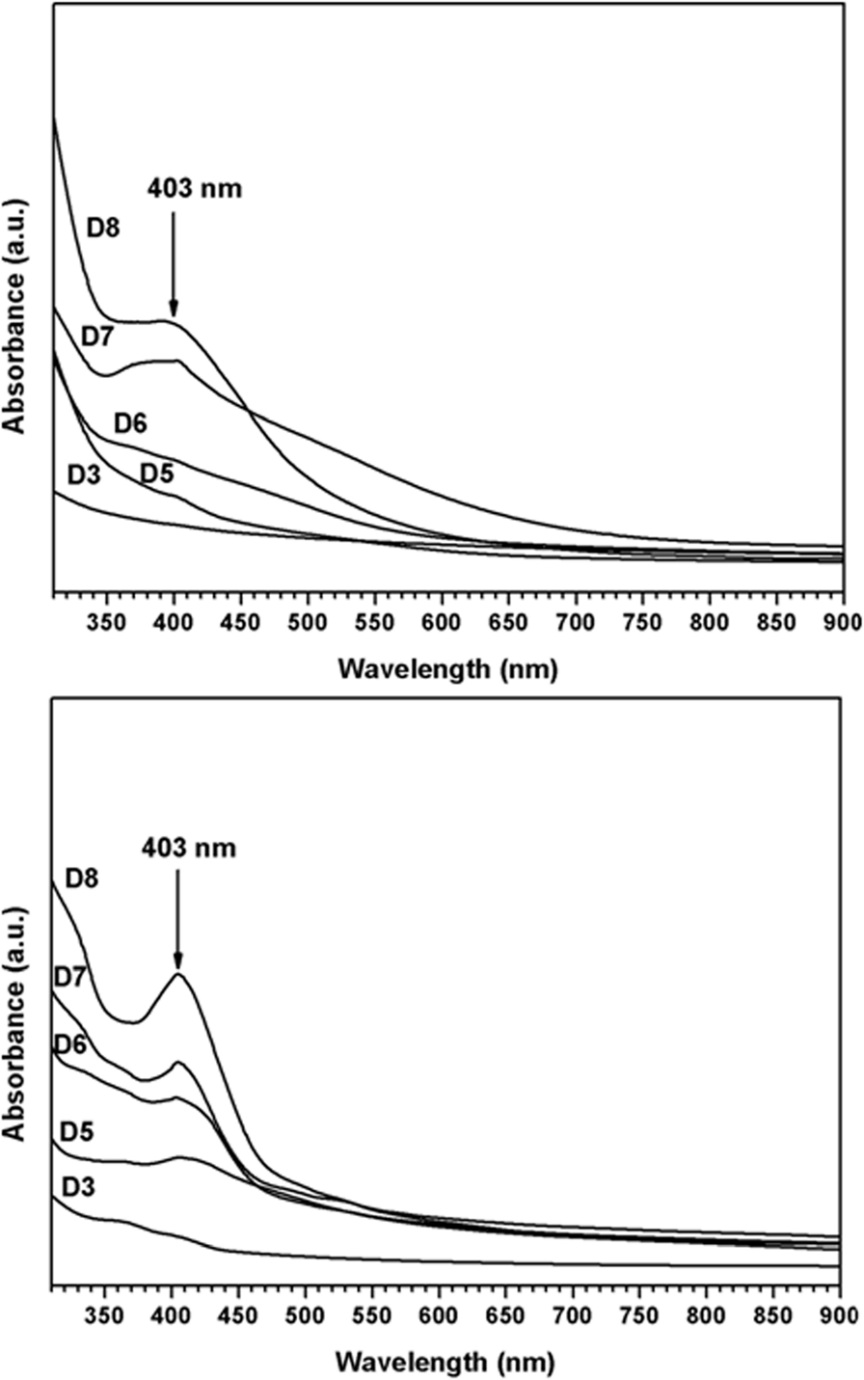
Time-dependent UV spectra of silver nanoparticles prepared using the ECM of *Streptomyces spiralis* (upper figure) and *Streptomyces rochei* (lower figure), D# represents the day on which Streptomyces strain was filtered an extracellular matrix was extracted and used for AgNPs preparation

**Fig. 4:**
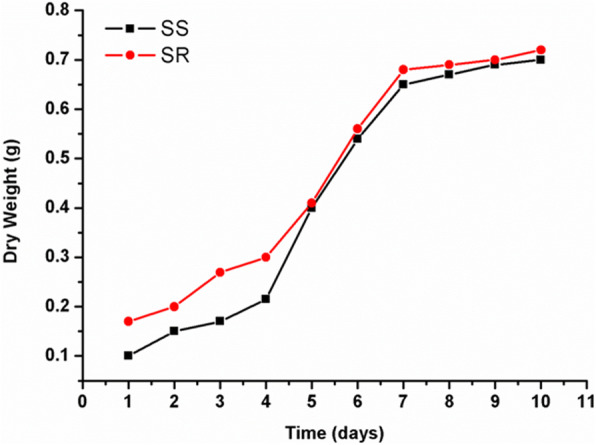
Growth curve of Streptomyces strains, SS represent *Streptomyces spiralis*, and SR refer to *Streptomyces rochei*

**Fig. 5:**
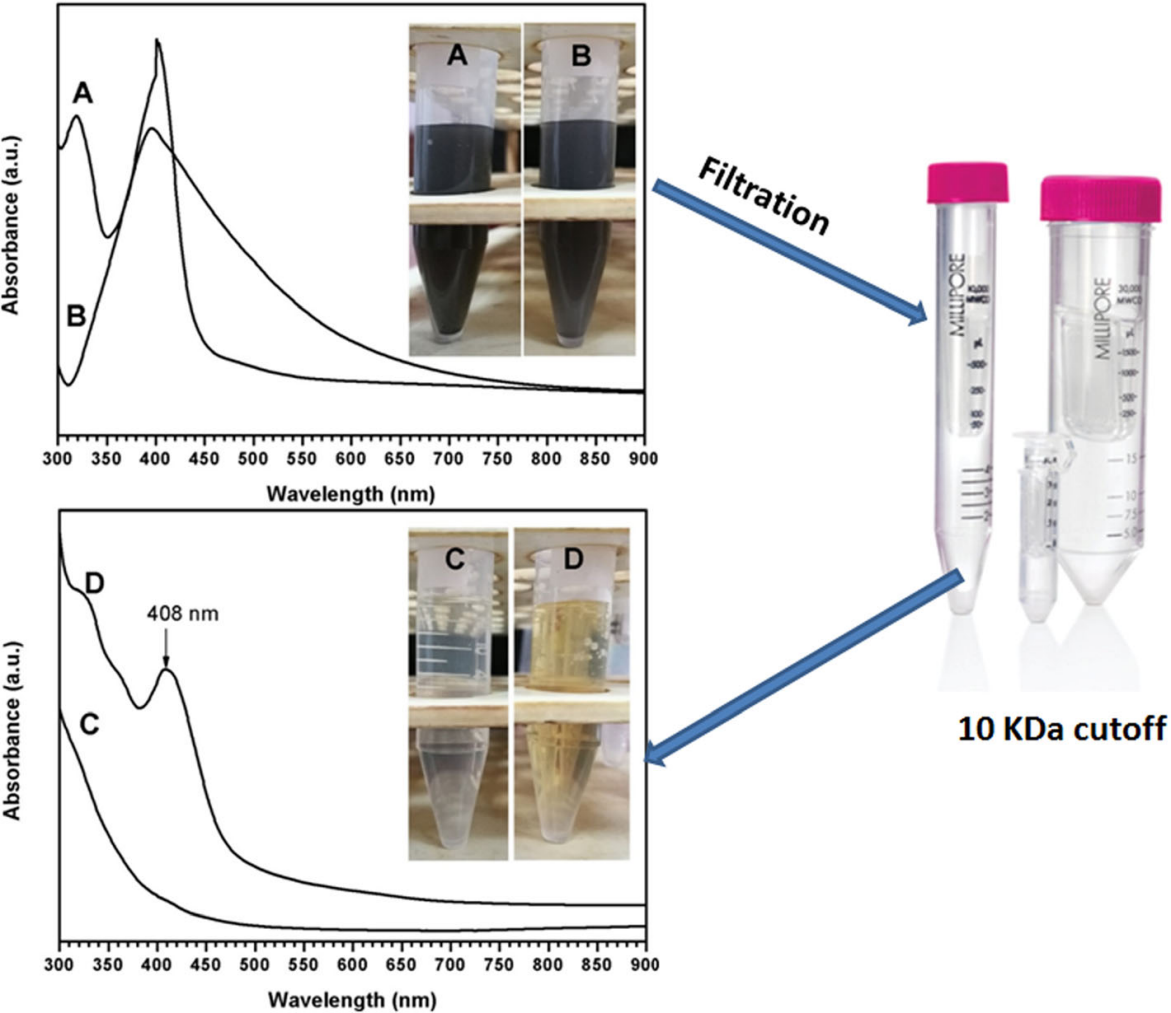
UV spectra of the concentrated samples of SS (**a**) and SR (**b**) in the upper image and UV spectra of the filtrates that passed through the filter for SR (**c**) and SS (**d**)

The investigated AgNPs scans were determined by UV-spectrophotometer as illustrated in Fig. 3. The incubation time was taken as AgNPs design factor in this measurement, in order to assess its effect on the resulted spectra of the surface plasmon resonance [34, 35]. It was observed that all the spectra possess main band around 403, which indicates the formation of AgNPs as early reported [35, 36]. This band was highly pronounced in late time of incubation (7, 8 days) and was very small and hardly detected in early stages of incubation (3–5 days). Thus, it is thought that the increase of time allowed higher reduction of silver nitrate solution by the investigated strains. It is worth highlighting that, *Streptomyces rochei* starts reduction of silver nitrate in D3 (as small band could be detected at this stage) earlier than *Streptomyces spiralis*, which stars the reduction process at D5 (Figs. 4 and 5).

### Growth curve of selected Streptomyces isolates

Maximum AgNPs production was observed on eighth day of incubation. Further increase in incubation time resulted in gradual decrease in production of nanoparticles which reversed to the growth curve of Streptomycetes strains as shown in Fig. 4 the dry weight increased with increasing in incubation days. It can be noted that the strain type possesses no remarkable change in their growth rates; they were almost identical especially starting from the 5th day.

### SDS-PAGE of the extracellular matrix protein of SS and SR stains

**Fig. 6:**
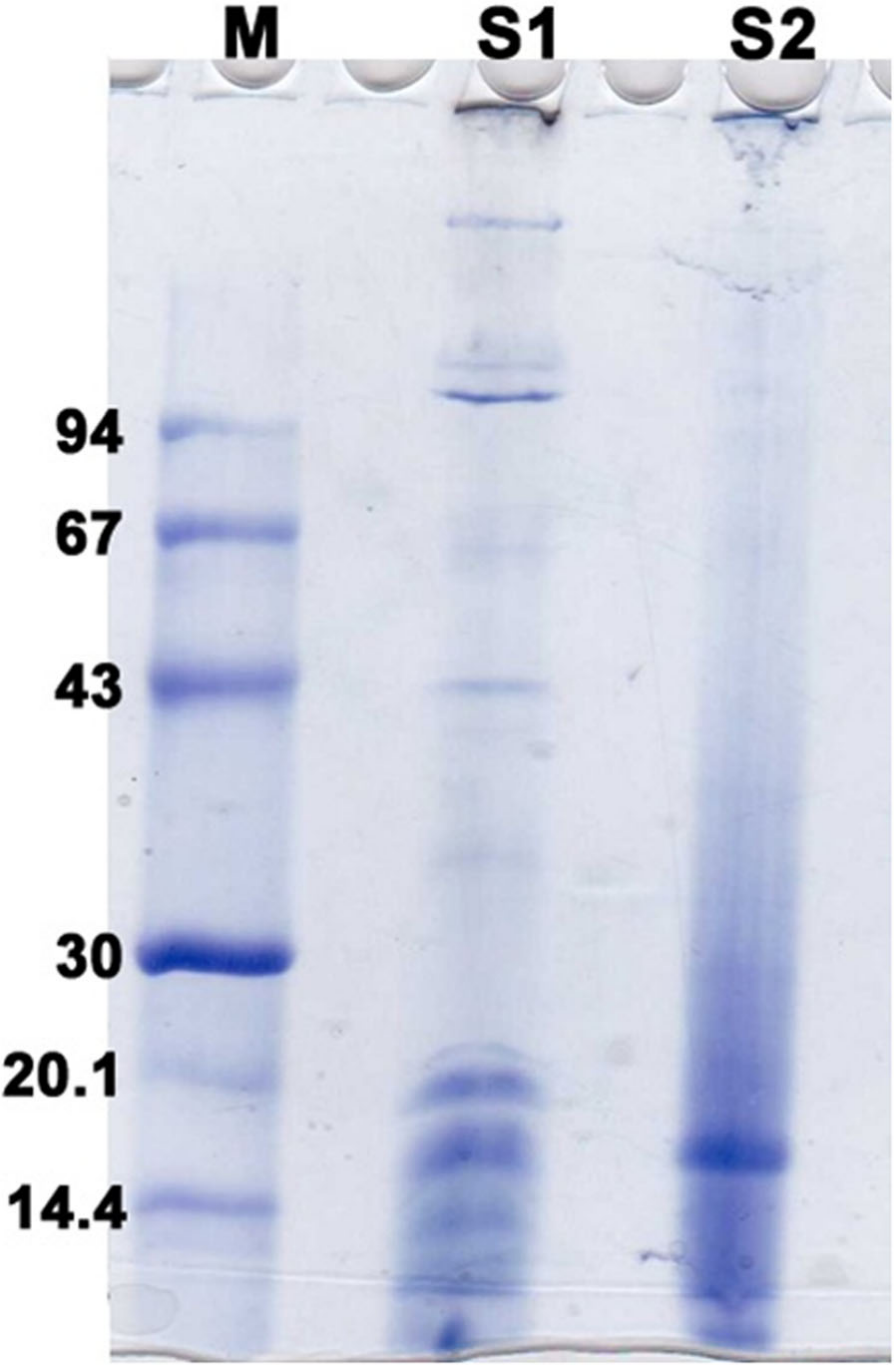
Proteins bands identified by SDS-PAGE in the concentrated extracellular matrix of Streptomyces broth, (M) Marker (lane 1), (S1) *Streptomyces spiralis* (lane 2), (S2) *Streptomyces rochei* (lane 3)

Figure 6 shows that the bands of the extracellular proteins in both actinomycetes strains are diverse in their molecular sizes. Interestingly, there is an observable correlation between the diversity of the extracellular proteins of each strain and the polydispersity of biogenic silver nanoparticles. In *Streptomyces spiralis*, several bands were observed ranging from low to medium molecular weights which is correlated well with the broad UV band of Ag NPs and TEM images that indicate their high polydispersity. In contrast, the UV and TEM analyses of Ag NPs prepared by the extract of *Streptomyces rochei* showed narrow band width and narrow size distribution, respectively, which might be due to the detection of less extracellular protein bands in SDS-PAGE analysis. Therefore, it can be suggested that the number and diversity of proteins secreted by Streptomycete strains is tightly correlated with the polydispersity of the biogenic nanoparticles prepared in the broth of Streptomycetes.

### Particle size and distribution by TEM

**Fig. 7:**
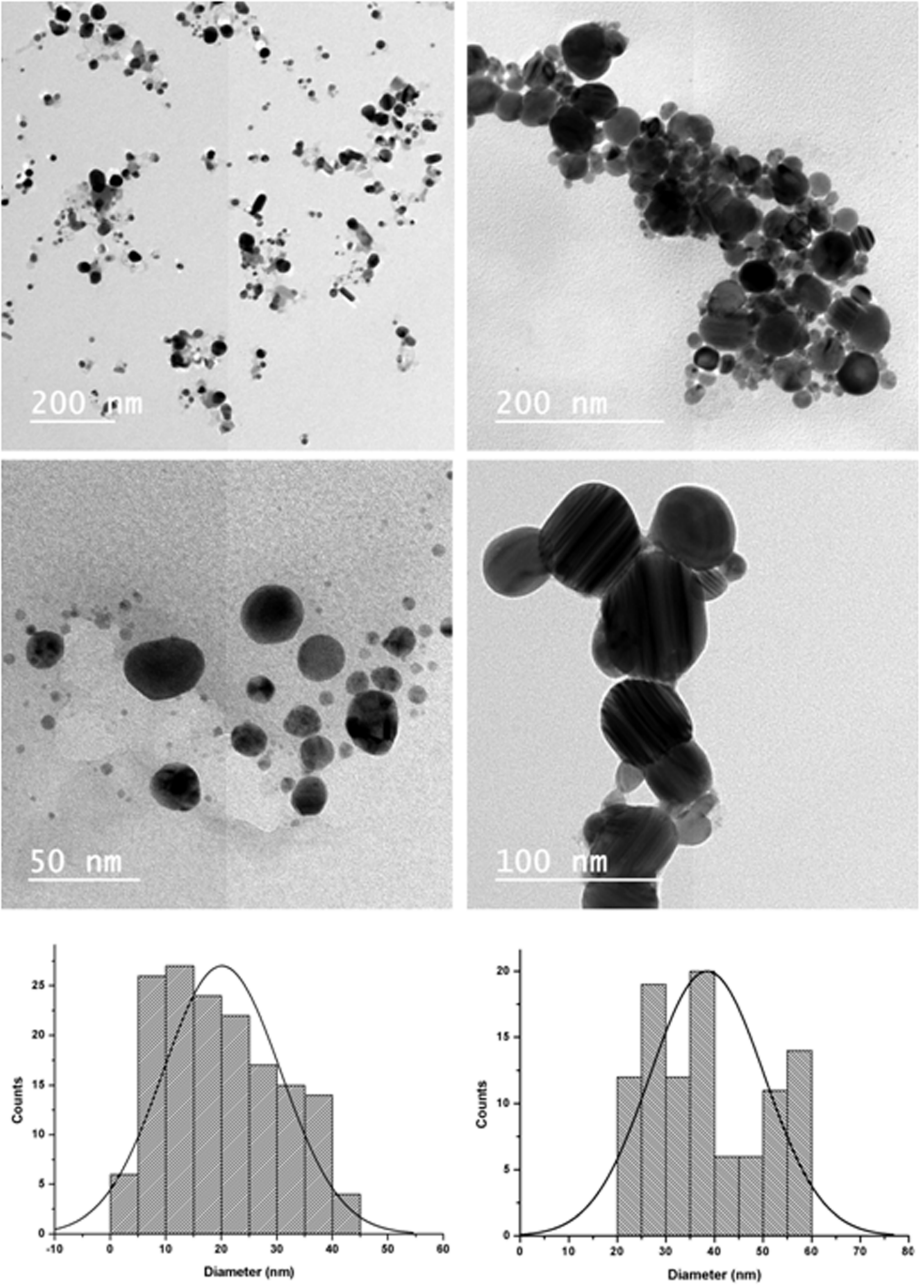
Left column represents AgNPs prepared by *Streptomyces rochei* and right column is assigned to the AgNPs prepared by *Streptomyces Spiralis*

Shape and diameter of the AgNPs prepared by the Streptomyces strains were evaluated by TEM as demonstrated in Fig. 7. The AgNPs prepared by *Streptomyces rochei* (the left column of Fig. 7) showed spherical nanoparticles with diameter range of 5–40 nm and maximum particle diameter distribution of 20 nm. On the other side, the AgNPs prepared by *Streptomyces spiralis* (the right column of Fig. 7) showed spherical nanoparticles with diameter range of 20–60 nm and maximum particle diameter distribution of 40 nm.

**Fig. 8:**
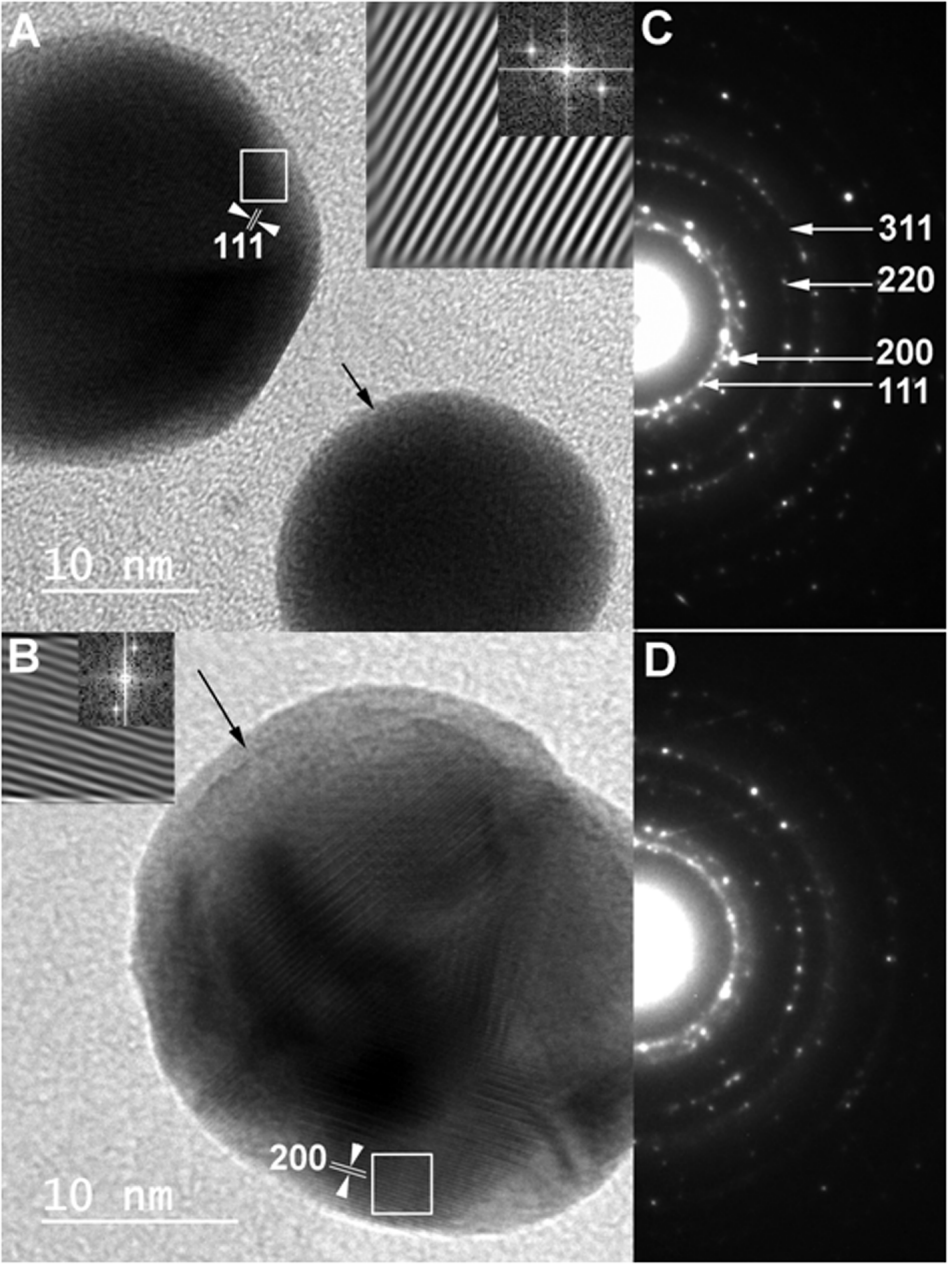
HR-TEM images and inset SAED of **a** and **c** AgNPs prepared by *Streptomyces rochei*; **b** and **d** of AgNPs prepared by *Streptomyces spiralis*

Moreover, HR-TEM images along with lattice spacing and SAED were recorded for AgNPs prepared by both strains as shown in Fig. 8. This was done to assess the whether the obtained NPs possess amorphous or crystalline nature. These assessments confirmed the crystalline nature of achieved AgNPs. Particularly, the recorded rings showed good arrangements of their illuminated spots for both samples that is confirming their crystalline owing to their ideal and typical arrangements.

### Absorption spectra of the AgNps

**Fig. 9:**
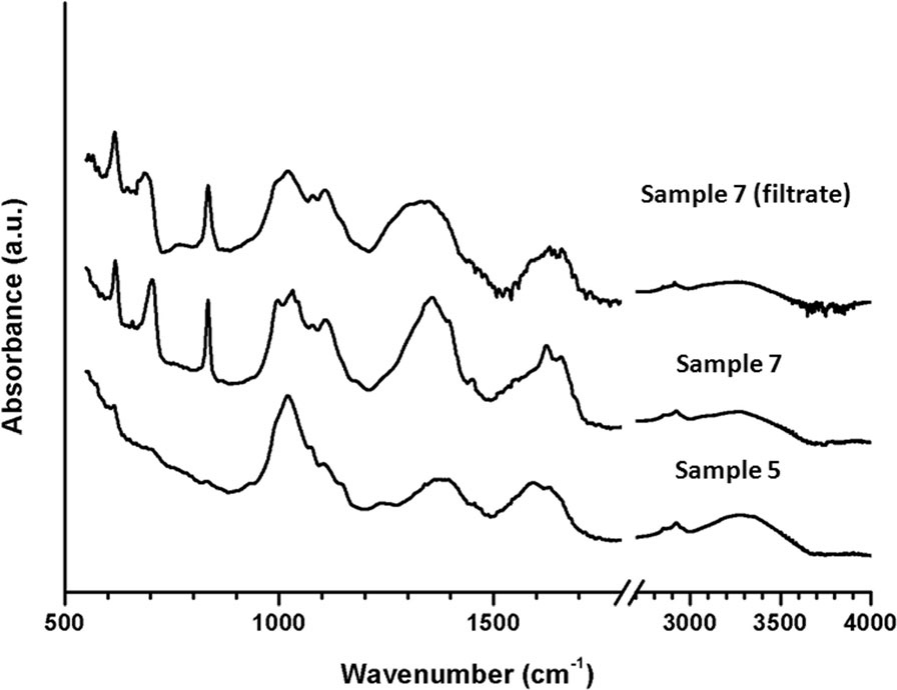
FTIR results of the obtained AgNPs by different strains before and after filtration by using an Amicon Ultra centrifugal filter with a 10 kDa cut-off

The absorption spectra were measured to introduce suggested interaction mechanism between the extracellular secretions and the AgNPs. The production of monodispersed AgNPs, capping, well-dispersed Ag-NPs in colloidal solution, stabilizing, and morphology are thought to be dependent on the utilized type of strain. In Fig. 9, it is noted that the all-absorption curves for the AgNPs and their filtrates (for strain *Streptomyces rochei*) showed several bands at 600, 690, 820, 990, 1030, 1013, 1392,1575, 1640, 2900, 3300, and 3325 cm^−1^. In details, the bands observed at 1640 cm^−1^ is assigned to amide I and amide II linkages of proteins, while band 3300 cm^−1^ is attributed for binding vibration of N–H groups. The band at 1575 cm^−1^ assigned to N–H stretching band of the amide I protein group. Band recorded at 1013 cm^−1^ are attributed to C–N stretching vibrations of aromatic and aliphatic amines. The C–H blending of aldehyde was recognized at band 1392 cm^−1^. The peak at 3325 cm^−1^ is assigned to the O–H stretching group of alcohol or N–H stretching of secondary amine. The bands located at 600, 690, 820, 990, 1030 cm^−1^were only observed in sample 7 and its filtrate, and these bands are thought to be specific for the extracellular secretions of *Streptomyces rochei*.

### Antibacterial activity of AgNPs against pathogenic microorganisms

**Fig. 10:**
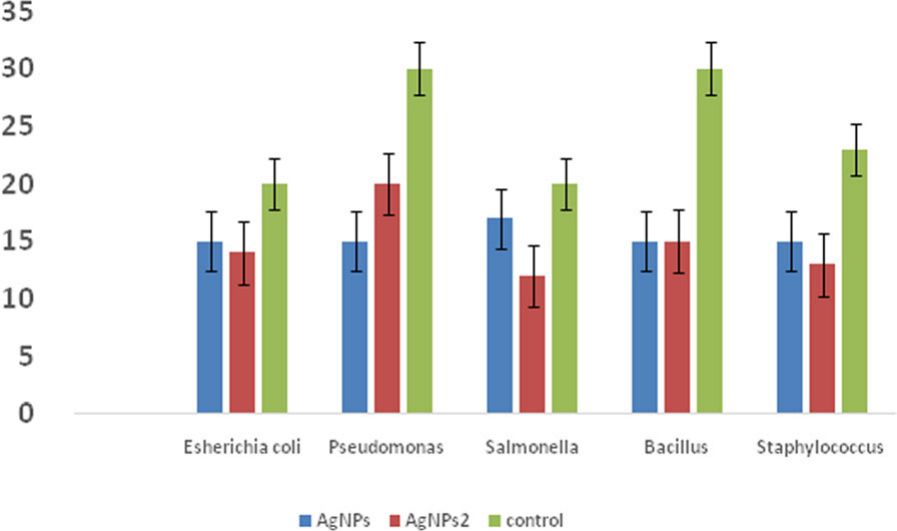
Antibacterial activity of biosynthesized AgNPs by two Streptomyces spp. against resistant bacteria

The antibacterial activity of AgNPs synthesis by two potent Streptomyces strain was performed. The results in Table 1 and Fig. 10 revealed that both gram-positive and gram-negative bacteria were inhibited by the produced AgNPs. The biosynthesized AgNPs by both Streptomyces species proved effective against the tested bacteria, but the inhibitory effect varied from one another. From *t* test table and at degree of freedom 2, it was shown that the calculated *t* values were significant.

**Table 1:** Antibacterial activity of biosynthesized AgNPs by two Streptomyces spp. against resistant bacteria. Values are mean of experiments performed in triplicate and data are expressed as mean ± SD

Test organism	Diameter of inhibition zone (mm)		
	AgNPs ***Streptomyces spiralis***	AgNPs ***Streptomyces rochei***	Control
*Escherichia coli*	15 ± 0.06	14 ± 0.15	20 ± 0.08
*Pseudomonas*	15 ± 0.16	20 ± 0.16	30 ± 0.3
*Salmonella*	17 ± 0.28	12 ± 0.1	20 ± 0.04
*Bacillus*	15 ± 0.05	15 ± 0.07	30 ± 0.1
*Staphylococcus*	15 ± 0.08	13 ± 0.05	23 ± 0.05

### Particle size and charges of AgNPs

**Fig. 11:**
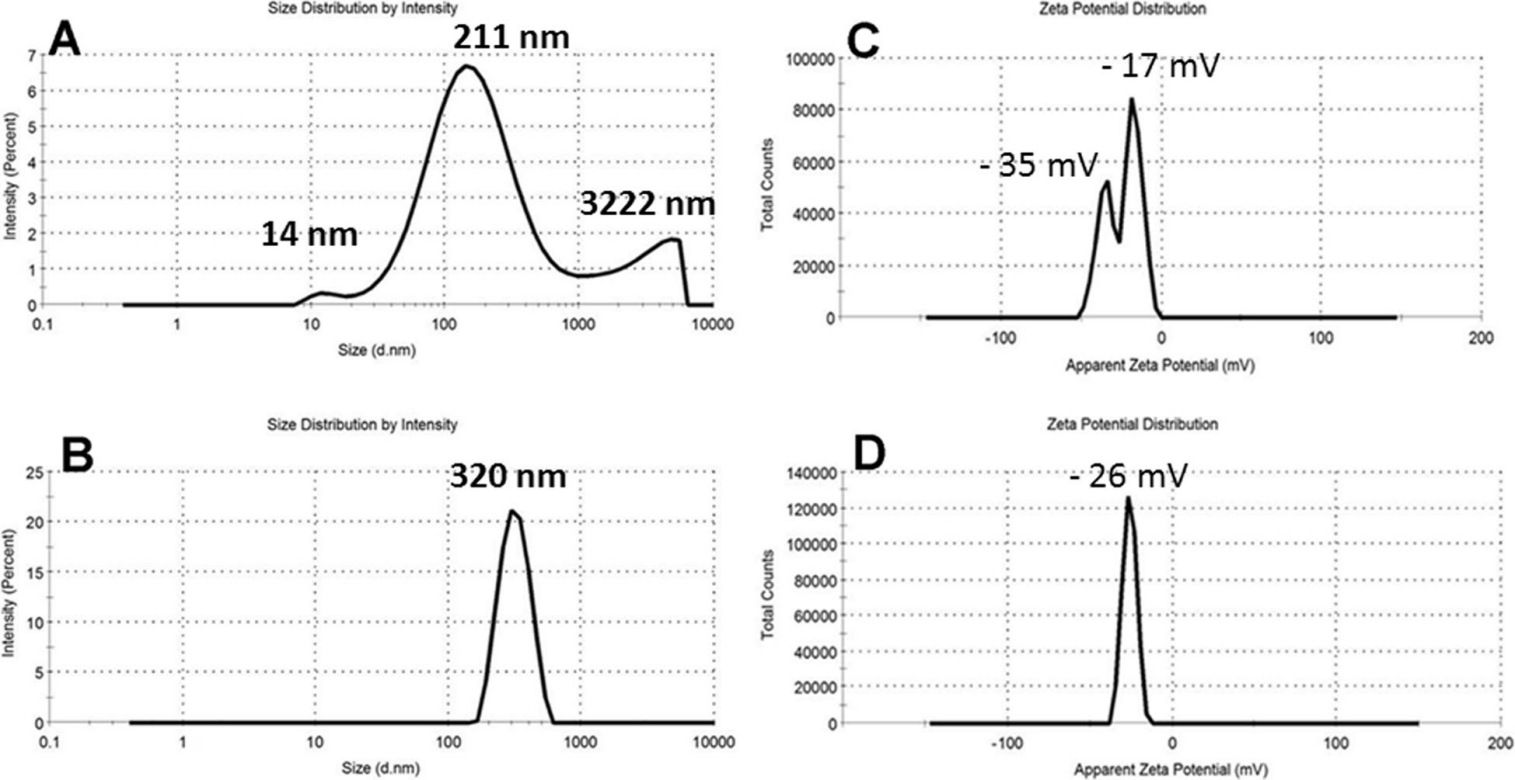
DLS measurements of sample 5 **a** particle size distribution, **c** zeta potential; sample 7 **b** particle size distribution and **d** zeta potential

Particles size was explored through DLS procedure by using solutions of AgNPs (0.1 mol/L) for each sample and they were found in the range from 200 to 350 nm. The higher diameter was recorded as 320 nm for sample 7 (Fig. 11b) with narrow range of particle diameters, thus indicates monodispersion behavior for the AgNPs achieved by *Streptomyces rochei*. On the other side, sample 5 (AgNPs prepared by *Streptomyces spiralis*) (Fig. 11a) introduced different particle size with very broad range of diameters 14, 211, and 3222 nm. These estimations for size of the AgNPs are different from TEM results, which could owe to the variation in the sample preparation technique for DLS and TEM. Zeta potential is a significant factor, which shows electrostatic forces among adjoining and comparable particles inside colloidal solution. The zeta likely qualities for biosynthesized AgNPs in this investigation were found to be − 26 mV for the AgNPs achieved by *Streptomyces rochei* (Fig 11d). While AgNPs prepared by *Streptomyces spiralis* (Fig. 11c) exhibited different two charges − 35 and – 17 mV. These results could be owed to different extracellular secretions by the two strains of Actinomyces [37].

## Discussion

Color changes during extracellular biosynthesis of AgNPs were previously reported in other studies [34, 35]. It was demonstrated that extracellular biosynthesis of NPs is mediated by different enzymes present on the microbial cell membrane or released to the growth medium [36]. Thus, the produced NPs may be adsorbed on the cell membrane or be present in the medium. The extracellular biosynthesis of NPs in microorganisms may occur under stress free conditions as a result of the presence of meta lions in the growth medium. In addition, it provides less laborious, economic, and large-scale NPs synthesis by easier processing [38]. Reductive proteins secreted by extracellular matrix of actinomycetes helps in direct synthesis of AgNPs and act as a capping agent to stabilize the size if the pointless proteins are avoided, and hence, it is reasonable for direct use in different applications. Therefore, after formation of silver nanoparticles, the solution was reduced in the volume and the proteins in the extracellular matrix were concentrated by using an Amicon Ultra centrifugal filter with a 10 kDa cut-off (Merck Millipore; Darmstadt, Germany).

It can be noted that sample A (*Streptomyces spiralis*; SS) exhibited two peaks at 320 and 400 nm and sample B (*Streptomyces rochei*; SR) showed one dominant peak with great intensity at 408 nm. After filtration, the spectrum of sample A is transformed into spectrum D and B is transformed into spectrum C. Spectrum C demonstrated no distinguished peaks for the AgNPs, while the spectrum D showed tow broad peaks 320 and 400 nm. These results suggest that the extracellular proteins SS and SR broths are different in their diversity, molecular sizes, and their ability to bind to silver nanoparticles. It has been reported that proteins can be used to stabilize the size distribution of nanoparticles by acting as a capping agent [39]. Early reported studies detailed the production of greater AgNPs a 63.14 nm and 88 nm for those synthesized by *Streptomyces* spp*.* [40] and *Phomagardeniae* [41], separately. Additionally, the action of biosynthesized AgNPs was associated with their size which improves their biocompatibility and strength [42]. This information can reason that, the microbial species have the limit with respect to biosynthesis of finer Ag-NPs as analyzed in the recently revealed NPs. In addition, it is though that the extracellular proteins secreted by *Streptomyces rochei* tend to produce relative monodispersed AgNPs compared to those produced by *Streptomyces spiralis*, which reveals the influence of the strain type on the size homogeneity of the produced nanoparticles. It is thought that the rings of selected area diffraction (SAED) represent are attributed to the 111, 200, 220, and 311 planes starting from inner to outer ring and has bright intense spots clearly indicating the face-centered cubic (fcc) structure of elemental silver [43, 44]. The particles stabilization, reduction, and distribution are essentially ascribed to the presence of proteins and phenols. These constituents react with AgNPs through different behaviors including amino groups, cysteine residuals, or by attraction because of negative charges of carboxylic groups secreted by various Actinomyces [45, 46]. In general, the gram-positive species are more vulnerable than gram-negative microorganisms due to having just an external peptidoglycan layer which is not a powerful penetrability boundary while the gram-negative species has an external phospholipidic film conveying the basic lipopolysaccharide component. In this manner, the bacterial cell membrane is impermeable to medications in gram negative species due to the presence of multi-layered of peptidoglycan and phospholipidic bilayer [47]. Undoubtedly, antibacterial activity of AgNPs is expected to possess different mechanisms compared to antibiotics [48]. Generally, the antibacterial property of AgNPs is essentially because of the delivery of silver cations from AgNPs that works as source for these nanoparticles [49]. However, scientists have clarified the potential mechanisms of antibacterial activity of silver nanoparticles including 1-AgNPs bounds to cell membrane of bacteria by electrostatic attraction and trouble the cell penetrability and cell breath because of the formation of oxygen species. 2-AgNPs attach to thiol groups of DNA and RNA, and influence the protein formation of bacteria [50, 51]. 3-Silver nanoparticles structure the pits on the cell surface and prompt the proton spillage bringing about cell passing [52]. The antimicrobial activity of AgNPs relies upon the size and state of the nanoparticles. Finer particles having the bigger surface area accessible for connection, which will give more bactericidal impact than the bigger particles and since they effectively, enter the cell membrane [22, 52–55]. It was noticed that *Streptomyces rochei* produced relatively monodispersed AgNPs due to the presence of single low molecular weight protein in its extracellular environment. While the diverse proteins in molecular sizes of *Streptomyces spiralis* contributed to the polydispersity in the sizes of Ag NPs. There is no significant impact on the antibacterial properties of Ag NPs against different gram positive and gram-negative bacteria.

## Conclusion

The extracellular secretions of two different strains of Actionbacteria were utilized to biosynthesize and cape AgNPs from the same source (silver nitrate) using the same incubation conditions. Two different AgNPs were obtained using these stains based on their investigated features such as time of production, particle diameter, charge, and dispersion of the particle sizes. Pronounced effect on these vital features of AgNPs is attributed to the different extracellular secretions of two different strains.

## Data Availability

Upon request

## Abbreviations

AgNPs: Silver nanoparticles
UV: Ultra violet
SDS-PAGE: Sodium dodecyl sulphate-polyacrylamide gel electrophoresis
TEM: Transmission electron microscope
FTIR: Fourier-transform infrared spectroscopy
DNA: Deoxyribonucleic acid
NADH: Nicotinamide adenine dinucleotide
PCR: Polymerase chain reaction
Taq: *Thermus aquaticus*
BLASTN: Basic Local Alignment Search Tool
NCBI: National Center for Biotechnology Information
HR-TEM: High-resolution transmission electron microscope
DLS: Dynamic light scattering
SD: Standard deviation
